# Recent advances in the cleavage of non-activated amides

**DOI:** 10.3762/bjoc.22.23

**Published:** 2026-02-19

**Authors:** Eun-Sol Choi, Hyo-Jun Lee

**Affiliations:** 1 Department of Chemistry, Kunsan National University, Gunsan 54150, Republic of Korea

**Keywords:** amide, amide activation, esterification, non-activated amide, transamidation

## Abstract

The amide bond is one of the most fundamental and widely utilized functional groups in organic chemistry, central to the structures of pharmaceuticals, bioactive molecules, and advanced materials. However, its exceptional resonance stabilization renders the C–N bond highly inert, posing a persistent synthetic challenge for its transformation. While twisted amides with distorted C–N bonds have offered useful reactivity enhancements, the selective activation of conventional, non-activated amides remains far more difficult. This review summarizes recent advances over the past decade in the activation and cleavage of non-activated amide C–N bonds for their conversion into diverse carboxylic acid derivatives. Key strategies covered include transition-metal catalysis, electrophilic activation, strong base-counter-cation systems, and *N*-based activating groups that enable chemoselective bond cleavage. Together, these developments provide powerful tools for amide functionalization and offer new opportunities for efficient, practical, and selective syntheses.

## Introduction

Serving as a cornerstone in organic chemistry, the amide bond is a fundamental and extensively utilized functional group, playing a central role in natural products, biomolecules, and functional materials. Its characteristic hydrogen-bonding ability strongly influences molecular conformation and intermolecular interactions, while the exceptional thermodynamic stability of the amide moiety contributes to its persistence in diverse atmospheric and biological environments [1–5]. Owing to these valuable features, amide derivatives are ubiquitous across the pharmaceutical, agrochemical, dye, polymer, and renewable-energy industries [6–11]. Accordingly, the development of efficient methods for both amide-bond formation and the selective transformation of amide functionalities remains a major objective in contemporary synthetic chemistry [12–16].

**Scheme 1:**
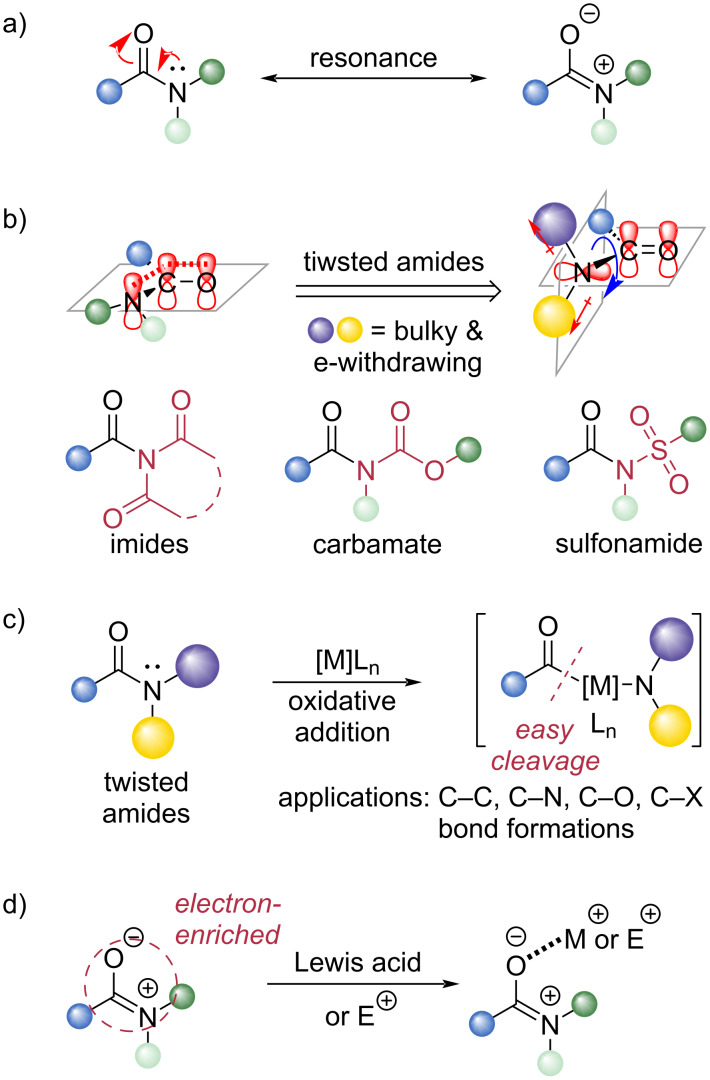
a) Resonance structure of amide. b) Concept of twisted amides. c) Transition-metal-catalyzed activation of twisted amides. d) Concept for activation of non-activated amides.

Despite their ubiquity, amides are notoriously unreactive toward classical acyl-substitution pathways. This inertness arises from a strong amidic resonance, which confers partial C=N double-bond character and a planar geometry to the amide unit (Scheme 1a) [17–19]. As a result, nucleophilic attack at the carbonyl is disfavored, and even when a tetrahedral intermediate forms, collapse with expulsion of an amide anion is thermodynamically challenging. Consequently, harsh conditions such as high temperatures or strongly activating reagents are typically required to cleave the amide C–N bond for its chemical transformation [20–23].

To address this reactivity issue, twisted amides have emerged as a powerful solution [24–27]. Incorporation of sterically demanding and electron-withdrawing substituents on the nitrogen (e.g., tosyl, carbamate, or acyl groups) distorts the planarity of the amide and diminishes amidic conjugation (Scheme 1b). These “twisted” amides exhibit a dramatically enhanced electrophilicity at the carbonyl carbon and a significantly weaker C–N bond strength [28–31]. Such activation has enabled a wide array of transformations, including metal-catalyzed cross-couplings via oxidative addition into the amide C–N bond (Scheme 1c) [32–36], and transition-metal-free acyl substitutions facilitated by improved leaving-group ability [37–40]. Although twisted amides have revolutionized amide-bond activation, their preparation often requires multistep synthesis or the installation of strongly electron-withdrawing groups, thereby limiting their broad applicability. Thus, the selective activation and transformation of non-twisted, non-activated amides remains a significant and persistent challenge in synthetic chemistry [41–44].

Classical approaches for activating non-activated amides typically rely on transition-metal catalysts acting as Lewis acids, which coordinate to the carbonyl oxygen and enhance electrophilicity (Scheme 1d). However, such methods often exhibit limited substrate scope, generally restricted to amides with low steric hindrance, such as DMF or DMAC. In recent years, several new strategies have emerged to overcome these limitations: (i) transition-metal-free activation using electrophilic reagents, (ii) strong-base-promoted transformations enhanced by alkali-metal counter-cation effects, and (iii) installation of activating groups on the nitrogen to enable chemoselective cleavage under tailored conditions. These approaches collectively offer promising avenues for the efficient transformation of non-activated amides under increasingly mild and practical conditions.

In this review, we summarize key advances from the past decade in the activation, cleavage, and transformation of non-twisted, non-activated amides. Each class of methodology is discussed in terms of its underlying activation concept, representative substrate scope, mechanistic features, and unique advantages or limitations, with the goal of providing a comprehensive overview of current strategies and future opportunities for amide-bond manipulation.

## Review

### Transition-metal catalysis

**Scheme 2:**
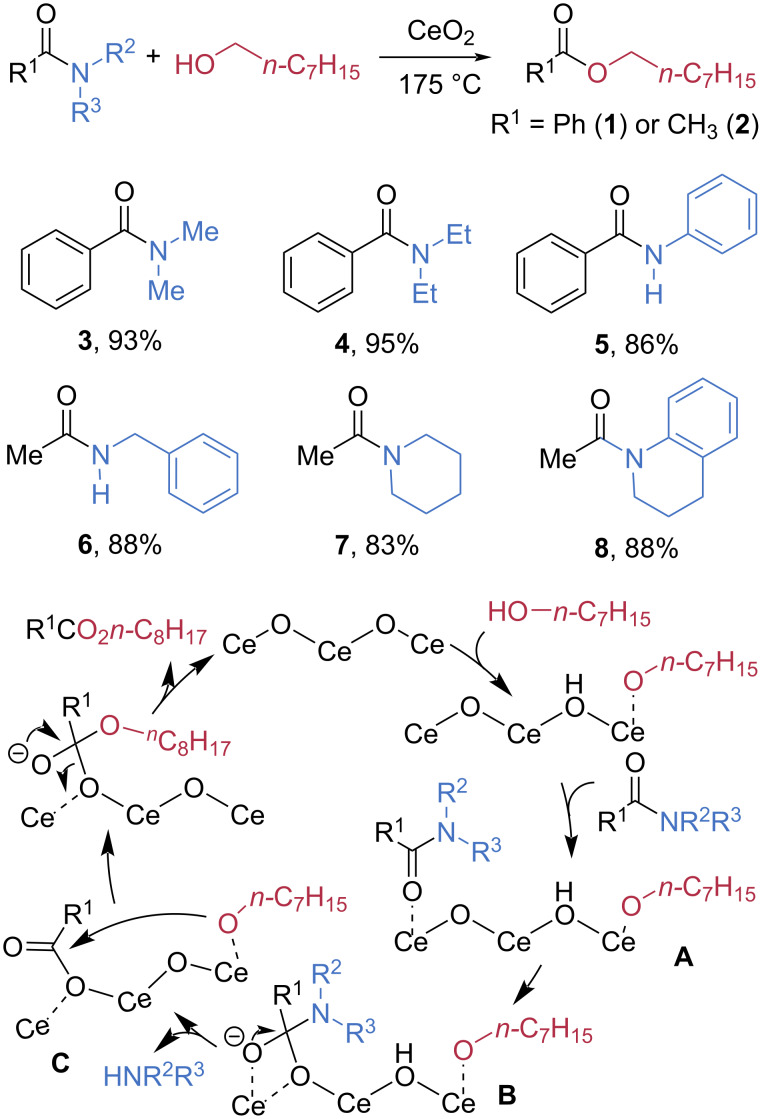
Esterification of amides catalyzed by CeO_2_.

Various transition metals that function as Lewis acids have been employed as reliable tools to activate amide bonds through coordination with the amide oxygen lone pair [45]. Although the substrate scope of such methods remains limited, several successful examples with hindered amides have recently been reported. In 2019, Shimizu et al. reported the esterification of secondary and tertiary amides with octanol catalyzed by CeO_2_, utilizing the advantage of its cooperative acid–base functionalities (Scheme 2) [46]. Under the optimized conditions, *N*,*N*-dimethylbenzamide (**3**) and *N*,*N*-diethylbenzamide (**4**) were smoothly converted to octyl benzoate (**1**) in 93% and 95% yield, respectively. *N*-Phenylbenzamide (**5**) also afforded **1** in 86% yield. Secondary and tertiary acetamides **6**–**8** were also successfully cleaved, affording the product **2** in high yields. The CeO_2_ catalyst was recyclable, although a slight decrease in yield was observed upon reuse. Notably, catalytic activity was fully restored after recalcination prior to reuse. Based on DFT calculations, the authors proposed that octanol is deprotonated and coordinated to Ce, while the amide substrate is simultaneously activated electrophilically through coordination to the Ce center (**A**). This dual activation enables the lattice oxygen of CeO_2_ to attack the carbonyl carbon, leading to cleavage of the C–N bond (**B**). Subsequent nucleophilic attack of the octoxide anion on the activated carbonyl center (**C**) then furnishes the ester product.

**Scheme 3:**
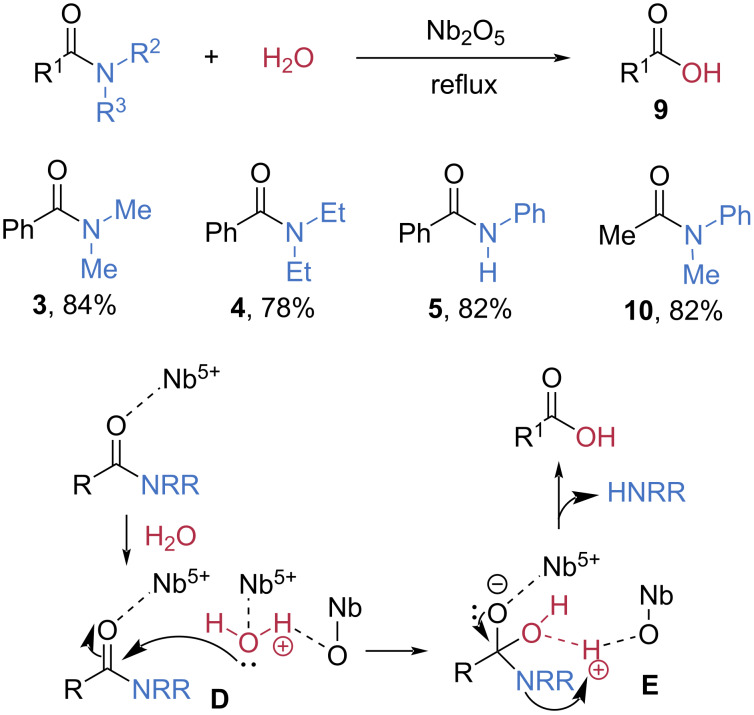
Hydrolysis of amides catalyzed by Nb_2_O_5_.

Later, the same group found that niobium could also serve as a heterogeneous Lewis acid catalyst for the hydrolysis of amides (Scheme 3) [47]. In the presence of a catalytic amount of Nb_2_O_5_, *N*,*N*-dialkylamides **3** and **4** and anilides **5** and **10** were efficiently converted into the corresponding carboxylic acids **9** in high yields, and the Nb_2_O_5_ catalyst could be successfully recycled. The authors proposed that coordination of the amide oxygen to the Lewis-acidic sites of Nb activates the amide toward nucleophilic attack. Simultaneously, water is pre-adsorbed on the basic oxygen sites of Nb_2_O_5_, enabling nucleophilic attack on the activated carbonyl center (**D**). Following amine release from the resulting intermediate **E**, the carboxylic acid is formed, regenerating the acidic and basic surface sites of the Nb_2_O_5_ catalyst.

**Scheme 4:**
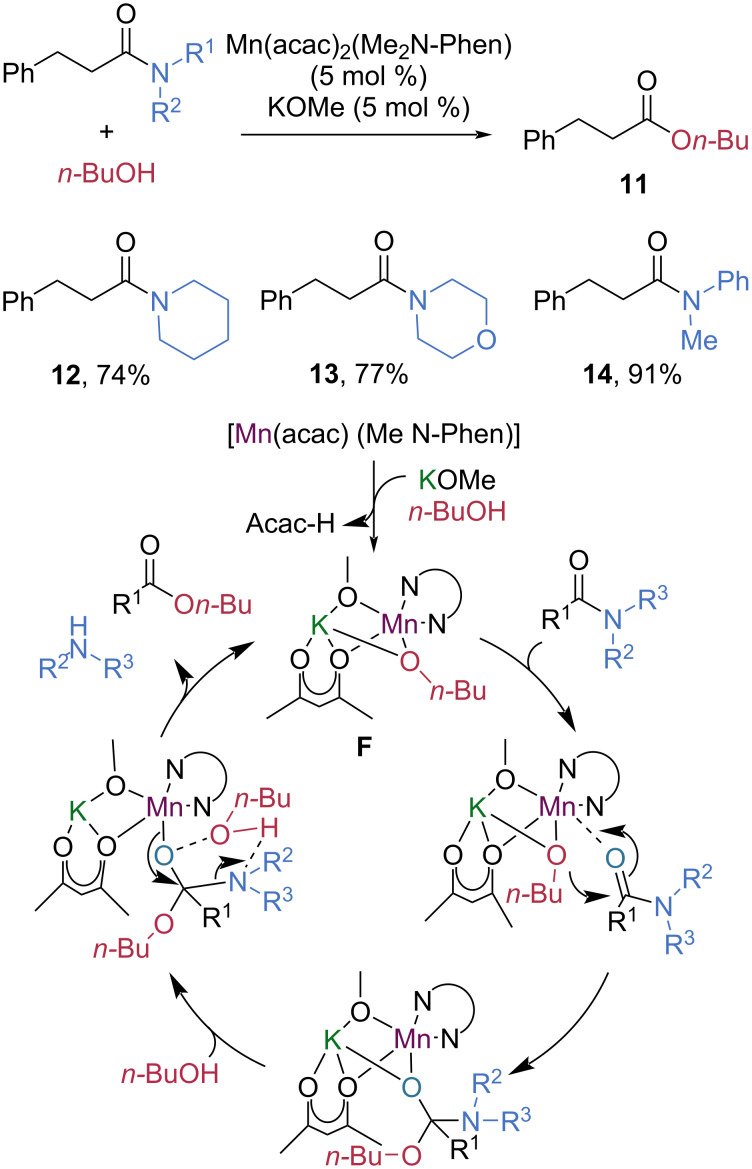
Manganese-catalyzed esterification of tertiary amides.

In 2020, Mashima et al. reported an intriguing accelerating effect of potassium alkoxides on the manganese-catalyzed esterification of tertiary amides (Scheme 4) [48]. They found that potassium methoxide significantly enhances the esterification process, thereby facilitating C–N bond cleavage in non-activated amides. In the presence of 5 mol % Mn(acac)_2_(Me_2_N-Phen) and potassium methoxide, using *n*-butanol as the nucleophile, tertiary amides **12**–**14** were converted to butyl 3-phenylpropionate (**11**) in good to high yields. The authors proposed that a manganese–potassium heterodinuclear alkoxylate complex **F**, formed from Mn(acac)_2_(Me_2_N-Phen), potassium methoxide, and *n*-butanol, serves as the key catalytic species. The dinuclear complex stabilizes the relevant transition states and intermediates, thereby lowering the energy barriers associated with both nucleophilic attack by *n*-butanol and subsequent C–N bond cleavage. As a result, the overall esterification process is significantly accelerated.

**Scheme 5:**
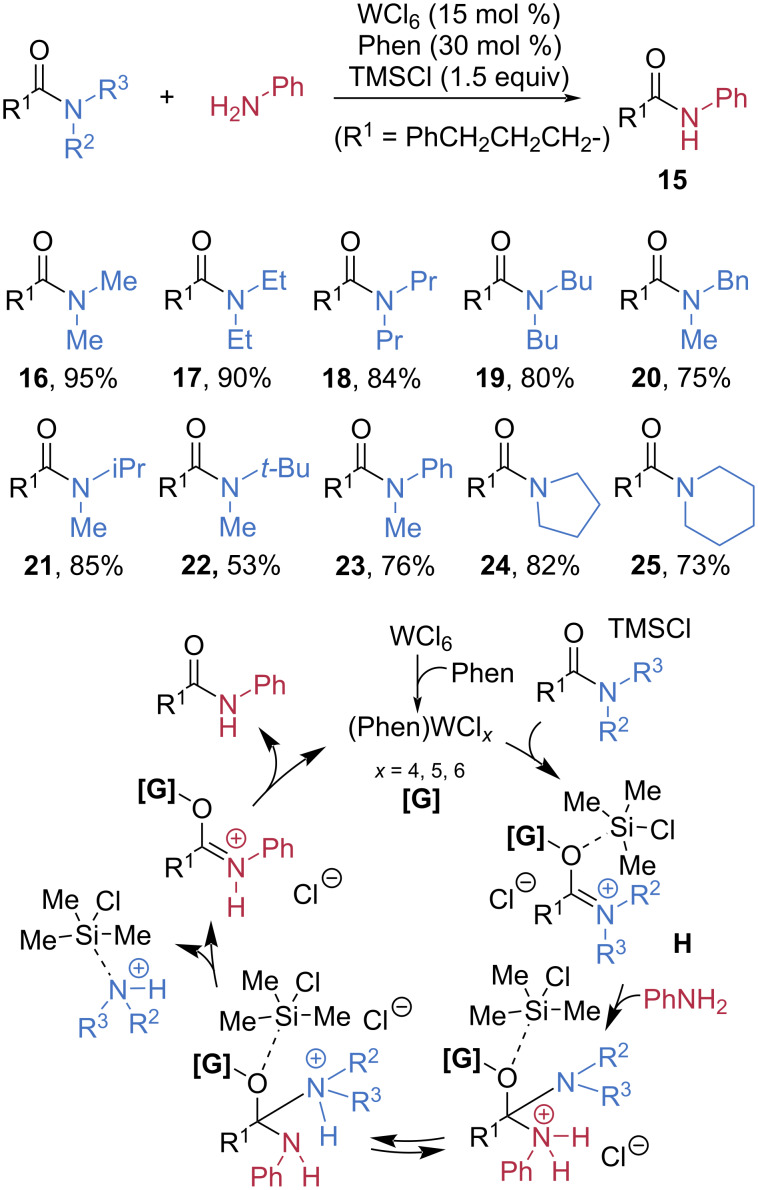
Tungsten-catalyzed transamidation of hindered tertiary amides.

In 2021, Ma et al. developed an elegant method for the transamidation of tertiary amides catalyzed by tungsten, exhibiting a broad substrate scope including hindered amides (Scheme 5) [49]. Using a catalytic amount of WCl_6_ in combination with 1,10-phenanthroline (Phen), various types of tertiary amides **16**–**25** were tested for the transamidation with anilines. All reactions furnished the anilide products **15** in good to high yields, and the results were highly dependent on the steric congestion of the substrate. With increased lengths of the alkyl substituent on the nitrogen, the yield was slightly decreased. Sterically hindered *N*-methyl-*N*-*tert*-butylamide **22** produced the corresponding anilide in only 53% yield. Mechanistically, WCl_6_ coordinates with Phen to generate the active tungsten species [**G**]. In cooperation with TMSCl as a Lewis acid, the catalyst activates the amide toward nucleophilic attack by forming an electron-deficient intermediate **H** that readily undergoes substitution.

**Scheme 6:**
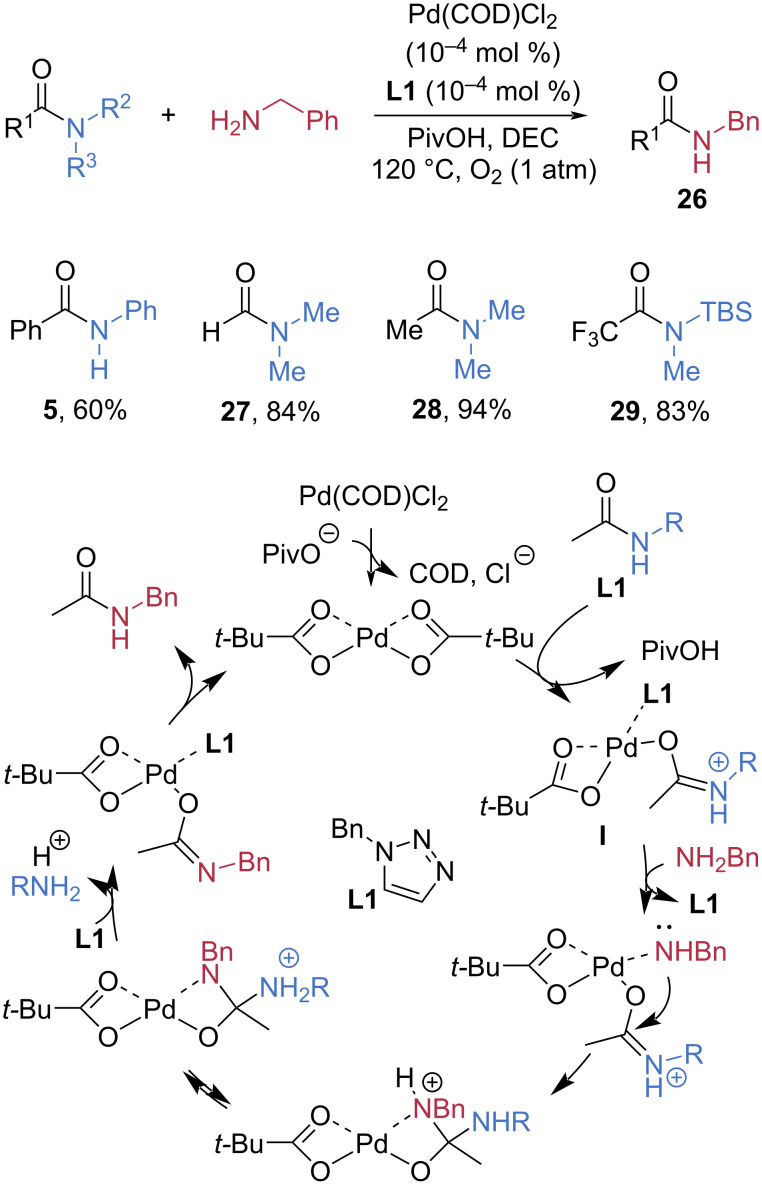
Palladium-catalyzed transamidation of amides.

Palladium complexes can also serve as efficient catalysts for activating amide C–N bonds. SanMartin et al. investigated a palladium-catalyzed transamidation of non-activated amides **5**, **27**–**29** with benzylamines, in which molecular oxygen acted as the palladium-activating agent (Scheme 6) [50]. Notably, high yields of the corresponding amide products **26** were obtained using only 10^−4^ mol % Pd(COD)Cl_2_ in combination with 1-benzyl-1*H*-1,2,3-triazole (**L1**). Based on kinetic curves, TEM images, catalyst poisoning experiments, MS identification of intermediates, and EPR spectra of the crude mixture, the authors proposed two kinetically distinct mechanistic pathways, a simplified version of which is described herein. The active catalytic species, palladium(II) pivalate, is formed in situ from Pd(COD)Cl₂ through ligand exchange with pivalic acid (PivOH). Complexation of this active Pd(II) species with ligand **L1** and the amide substrate generates a reactive intermediate **I**. Subsequent exchange of **L1** with benzylamine enables intramolecular nucleophilic attack, leading to transamidation.

### Transition-metal-free electrophilic activation

Since the keteniminium ion, which can be readily generated by electrophilic activation of enolizable tertiary amides, was investigated [51–52], it has been recognized as a useful and versatile intermediate for the modification and transformation of less reactive amides [53–54]. Recently, utilizing electrophilic activation, a transition-metal-free cleavage of the amide C–N bond has been developed as an environmentally benign approach under mild reaction conditions, exhibiting a broad substrate scope.

#### Esterification

**Scheme 7:**
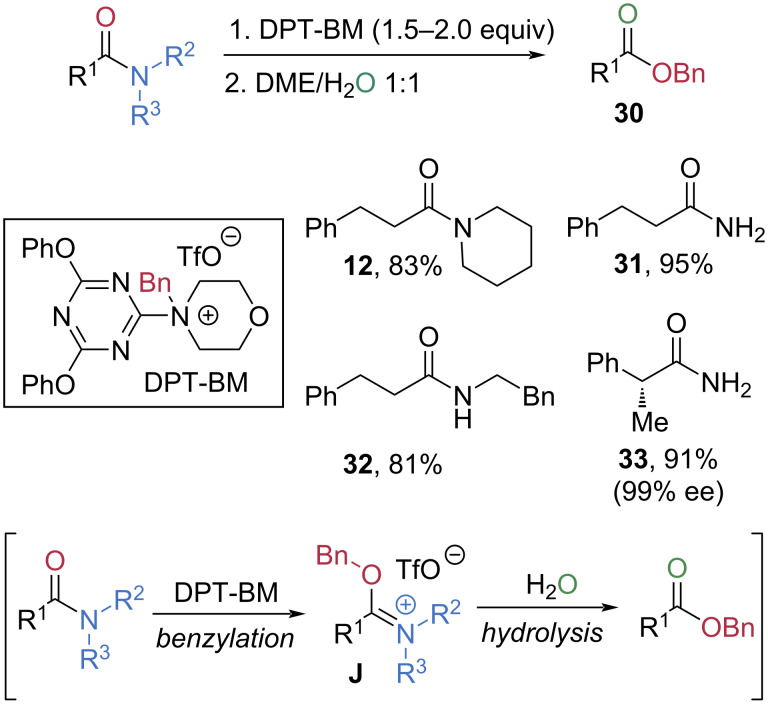
Synthesis of benzyl esters via electrophilic activation of amides using DPT-BM.

In 2016, the Kunishima group developed a mild esterification of amides using the triazine-based 4-(4,6-diphenoxy-1,3,5-triazin-2-yl)-4-benzylmorpholinium trifluoromethanesulfonate (DPT-BM) as an electrophilic benzylating reagent (Scheme 7) [55]. DPT-BM functions as an *O*-benzylating reagent that generates benzyl cation equivalents upon dissolution in the solvent under non-acidic conditions. Thus, the reaction of an amide with DPT-BM is proposed to form a benzyl imidate salt **J**, which subsequently undergoes hydrolysis to afford the benzyl ester **30**. Under the optimized conditions, primary, secondary, and tertiary amides **12**, **31**–**33** were successfully converted to benzyl esters in high yields. Notably, loss of enantiopurity of chiral amide **33** was not observed during esterification.

**Scheme 8:**
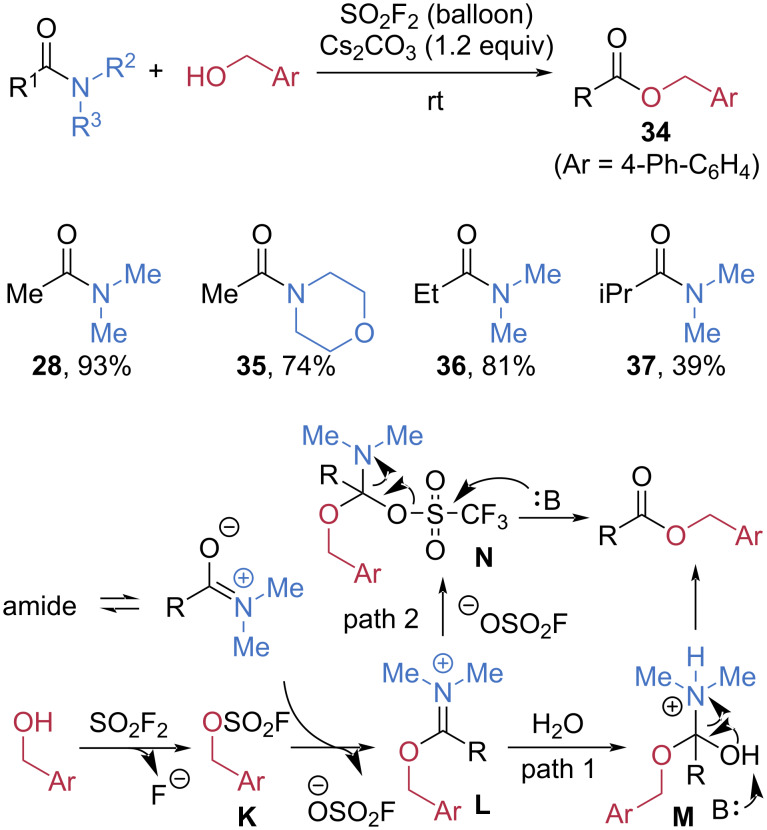
Esterification of amides promoted by SO_2_F_2_.

Sulfuryl fluoride (SO_2_F_2_) was demonstrated to be a powerful electrophilic reagent for amide-bond cleavage by Qin et al. (Scheme 8) [56]. Under an atmosphere of SO_2_F_2_, esterification of tertiary amides **28**, **35**–**37** smoothly afforded esters **34** in high yields, although the efficiency was reduced when a bulky substituent was introduced at the R^1^ position (**37**, 39% yield). The SO_2_F_2_-promoted esterification begins with the formation of fluorosulfate **K** through the reaction of the alcohol with SO_2_F_2_. This intermediate then undergoes an S_N_2 attack by the tautomeric form of amide, generating the iminium species **L**. In wet solvents (path 1), addition of water affords adduct **M**, which subsequently converts to the ester via deprotonation. In dry solvents (path 2), nucleophilic attack by the departing OSO₂F^−^ anion on **L** produces intermediate **N**, containing an S–O bond that is ultimately cleaved under basic conditions to yield the ester.

**Scheme 9:**
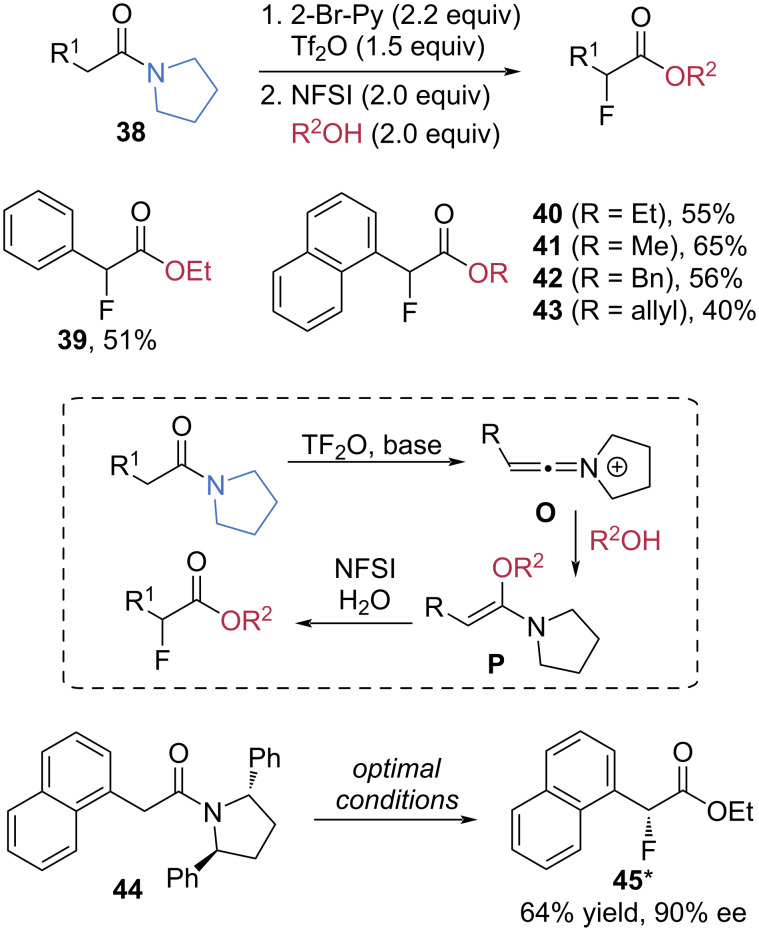
α-Fluorinative cleavage of pyrrolidine-based tertiary amides via double electrophilic activation with Tf_2_O and NFIS.

In 2021, the Evano group developed a double electrophilic activation strategy for tertiary amides using trifluoroacetic anhydride (Tf_2_O) and *N*-fluorobenzenesulfonimide (NFSI), enabling α-fluorinative C–N bond cleavage (Scheme 9) [57]. In the presence of 1.5 equivalents of Tf_2_O as an electrophile and 2.2 equivalents of 2-bromopyridine as a base, pyrrolidine-derived amides **38** were effectively activated. Subsequent addition of NFSI as the electrophilic fluorinating reagent, along with alcohols as the nucleophile, afforded the corresponding esters **39**–**43** in moderate yields. Mechanistically, the electrophilic activation of pyrrolidine-derived amides with Tf_2_O and 2-bromopyridine generates a keteniminium ion **O**, which undergoes nucleophilic addition to form an enamine intermediate **P**. Subsequent electrophilic α-fluorination followed by hydrolysis produces the α-fluoroester products. Notably, the *C*_2_-chiral amide **44**, incorporating (2*S*,5*S*)-2,5-diphenylpyrrolidine as a chiral auxiliary, furnished the enantioenriched ester 45* in 64% yield with 90% ee.

**Scheme 10:**
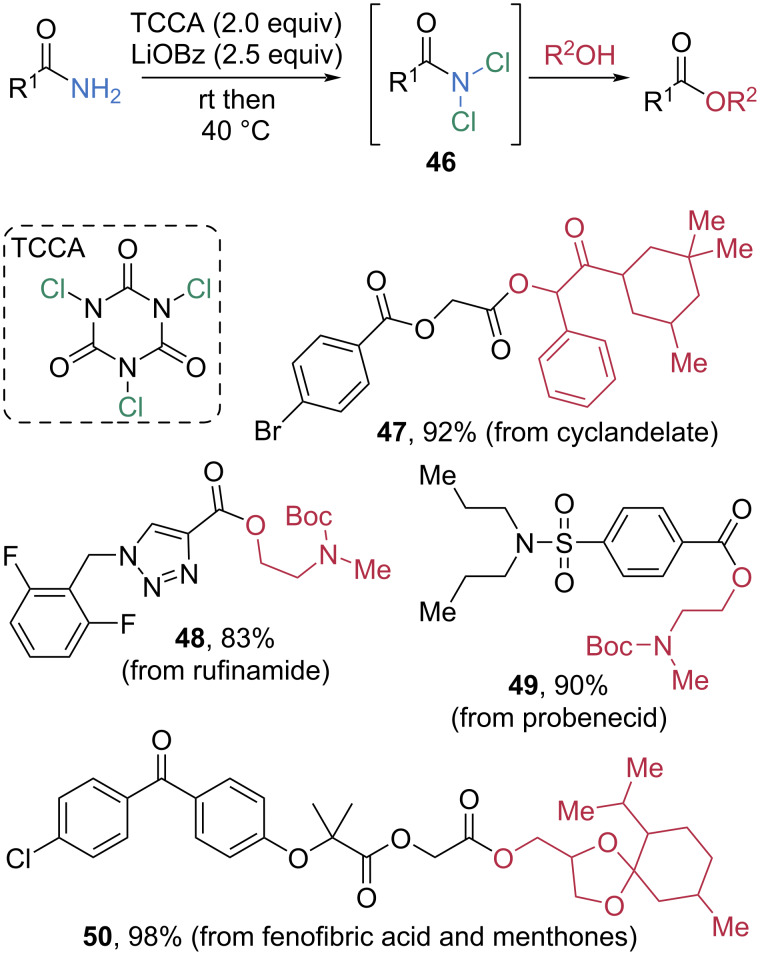
Esterification of primary amides using TCCA via the generation of RCONCl_2_.

Wan et al. investigated the chlorination of primary amides using trichloroisocyanuric acid (TCCA) to promote esterification (Scheme 10) [58]. In the presence of 2 equivalents of TCCA as a chlorinating reagent and 2.5 equivalents of lithium benzoate (LiOBz) as a base, primary amides are converted into the corresponding *N,N*-dichloroamide intermediates **46**, which then undergo smooth nucleophilic acyl substitution with alcohols to afford esters. The substrate scope of the esterification was indeed broad under mild conditions, enabling late-stage functionalization. For example, esterification of a fenofibric acid-derived primary amide with menthone derivatives furnished the corresponding ester **50** in 98% yield.

**Scheme 11:**
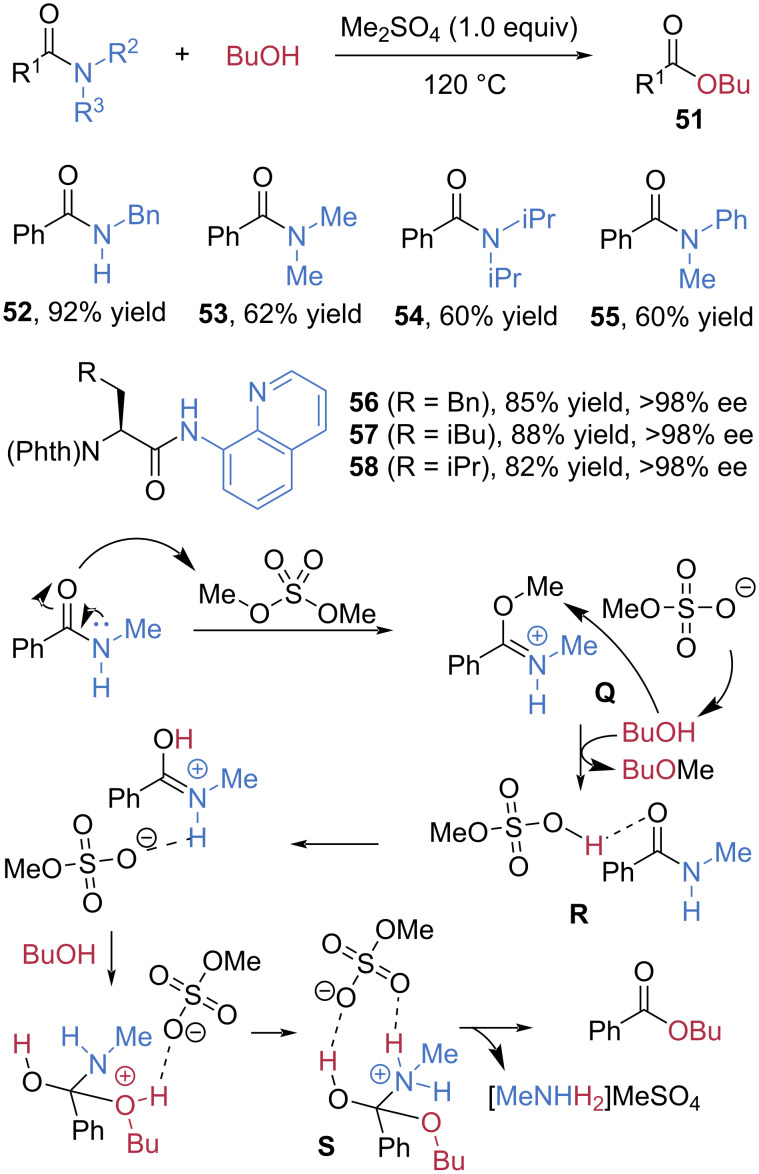
Esterification of amides via electrophilic activation with Me_2_SO_4_.

In 2024, Aisa et al. reported a facile and effective esterification of amides using dimethyl sulfate (Me_2_SO_4_) as a cheap and powerful coupling reagent (Scheme 11) [59]. In the presence of 1 equivalent of Me_2_SO_4_ at 120 °C, secondary *N*-benzyl benzamide (**52**) underwent direct esterification with butanol to afford butyl benzoate (**51**) in 92% yield. When *N,N*-disubstituted tertiary amides **53**–**55** were used, moderate yields were observed for the esterification. Notably, 8-aminoquinoline-derived chiral amides **56**–**58** were also smoothly cleaved in high yields without racemization. This is significant because the 8-aminoquinoline moiety is widely used in transition-metal-catalyzed asymmetric C–H activation reactions as a directing group, thus necessitating an efficient method for the cleavage of 8-aminoquinoline-derived amides. The role of Me_2_SO_4_ in this transformation was elucidated through DFT calculations. Methylation of the carbonyl oxygen by Me_2_SO_4_ generates the imidate-like intermediate **Q**. Subsequent nucleophilic attack of BuOH on the methyl group of **Q** leads to transition state **R**, accompanied by the departure of butyl methyl ether. Further attack of BuOH on the *O*-protonated amide, followed by an intramolecular proton transfer, furnishes transition state **S**, which is stabilized by two new hydrogen-bonding interactions. Finally, elimination of [MeNH_3_]MeSO_4_ delivers the butyl ester product.

**Scheme 12:**
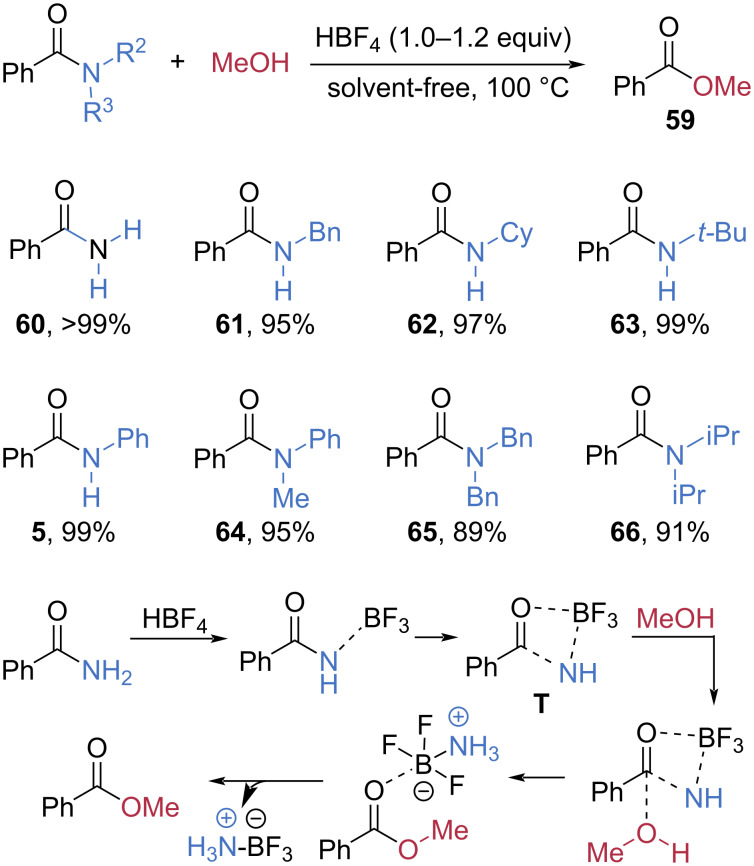
HBF_4_-mediated esterification of amides.

In 2025, Dou et al. developed a metal-free alcoholysis of amides mediated by HBF_4_, exhibiting a very broad substrate scope (Scheme 12) [60]. The esterification of benzamide (**60**) with methanol in the presence of 1 equivalent of HBF_4_ at 100 °C under solvent-free conditions afforded methyl benzoate (**59**) in quantitative yield. Secondary amides, including those possessing primary (**61**), secondary (**62**), and tertiary alkyl (**63**) and aryl moieties (**5**), were suitable substrates to produce **59** in excellent yields. Even extremely hindered tertiary amides **64**–**66** also furnished **59** in excellent yields. Mechanistic studies suggested that the amide NH_2_ group coordinates with the Lewis acid HBF_4_. Subsequent coordination between the carbonyl oxygen and boron generates intermediate **T**, which reacts with methanol to produce the ester product releasing ammonium boron trifluoride (NH_3_BF_3_).

**Scheme 13:**
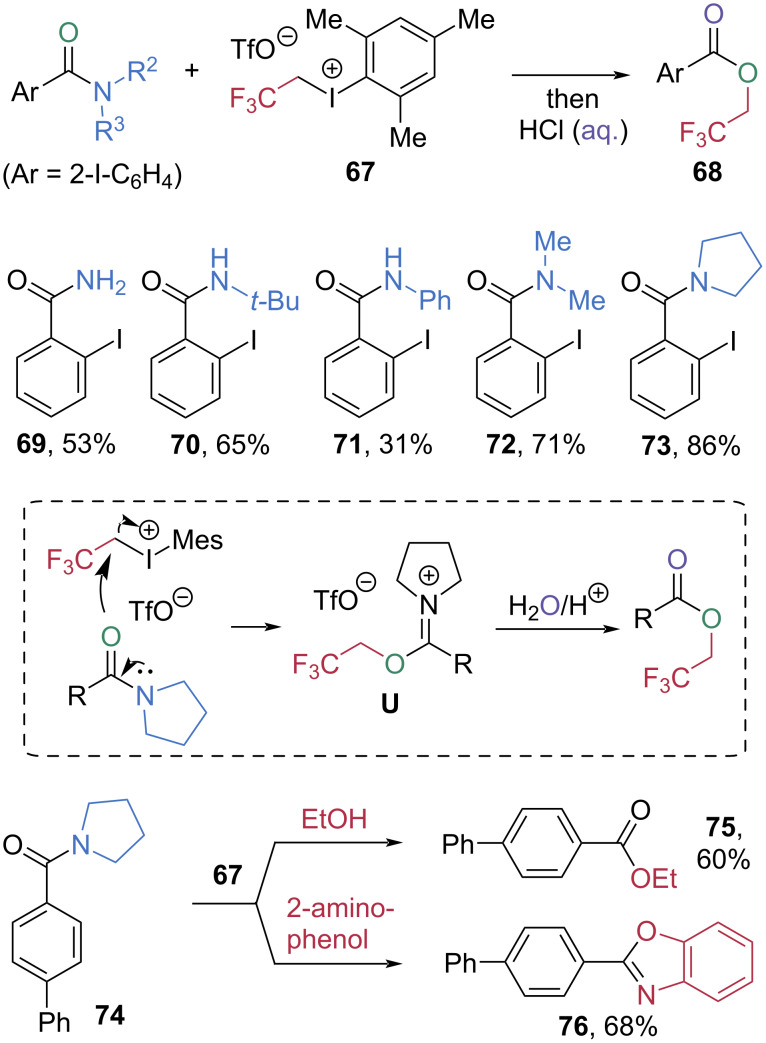
Synthesis of 2,2,2-trifluoroethyl esters via electrophilic esterification of amide promoted by **67**.

Prévost et al. developed 2,2,2-trifluoroethyl iodonium salt **67** as an electrophilic activator for amide C–N bond cleavage (Scheme 13) [61]. Using two equivalents of **67**, the esterification of primary and secondary 2-iodo-*N,N*-dimethylbenzamides **69**–**71** afforded 2,2,2-trifluoroethyl 2-iodobenzoate (**68**) in moderate yield. Higher yields were observed when tertiary amides **72** and **73** were employed for esterification. Among these, pyrrolidine-based amide **73** exhibited the highest efficiency, furnishing the ester **68** in 86% yield. Mechanistic studies indicated that the pyrrolidine-based amide **73** reacts with iodonium salt **67** to form an imidate triflate intermediate **U**, which was isolated and characterized by NMR and HRMS. Subsequent hydrolysis via water attack affords the trifluoroethyl ester. This strategy also enabled the formation of ethyl ester **75** and benzoxazole **76** when ethanol and 2-aminophenol were employed as nucleophiles, respectively.

**Scheme 14:**
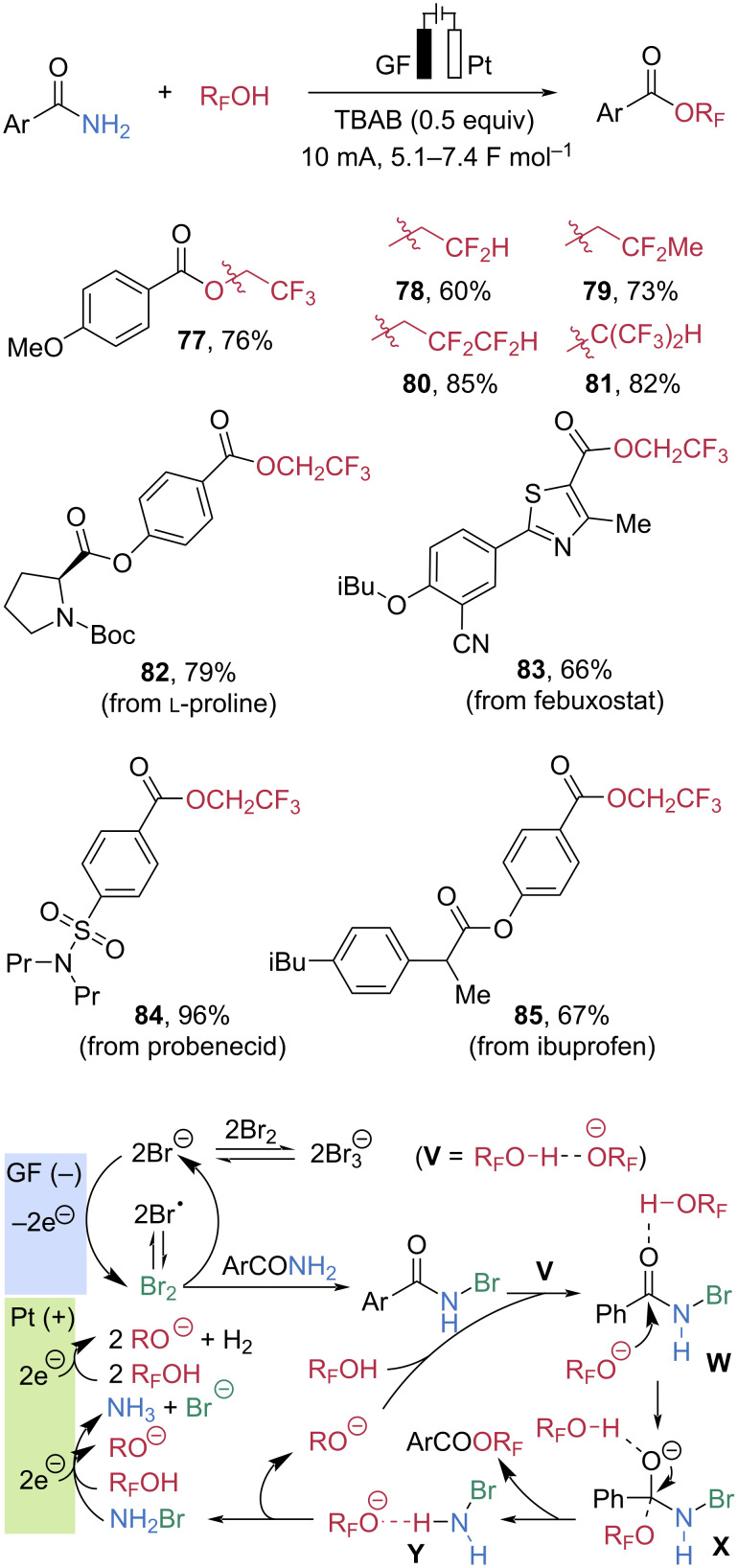
Electrochemical activation of C–N bonds for esterification.

Chen et al. recently investigated an environmentally benign approach to amide C–N bond cleavage using electrochemical activation (Scheme 14) [62]. In the presence of tetrabutylammonium bromide (TBAB) as the supporting electrolyte, the reaction of primary benzamides with trifluoroethanol (TFE) under constant current conditions (10 mA) furnished 2,2,2-trifluoroethyl benzoate **77** in 76% yield, employing a graphite felt (GF) anode and a platinum (Pt) cathode. Under the same conditions, several types of fluorinated alcohols were successfully employed as nucleophiles, generating the corresponding esters in good to high yields. Notably, the method displayed excellent chemoselectivity for primary amides. Therefore, multifunctional substrates derived from natural products or bioactive compounds underwent smooth esterification with TFE to give the corresponding 2,2,2-trifluoroethyl esters **82**–**85** in high yields. To elucidate the reaction mechanism, cyclic voltammetry experiments, control reactions, and DFT calculations were conducted. At the anode, bromide ions are oxidized to bromine (Br_2_), which converts the amide into *N*-bromobenzamide. Meanwhile, at the cathode, the alcohol is partially deprotonated to form an alkoxide, which engages in a stabilizing hydrogen-bonding interaction to produce the acid–base pair **V**. Nucleophilic attack of **V** on *N*-bromobenzamide forms a tetrahedral intermediate anion **X** via the activated transition state **W**. Subsequent reorganization of hydrogen bonding toward the nitrogen center promotes C–N bond cleavage to give the ester product and intermediate **Y**. Decomposition of **Y** regenerates **V** and produces NH_2_Br, which is reduced at the cathode back to bromide, completing the electrochemical cycle.

#### Transamidation

**Scheme 15:**
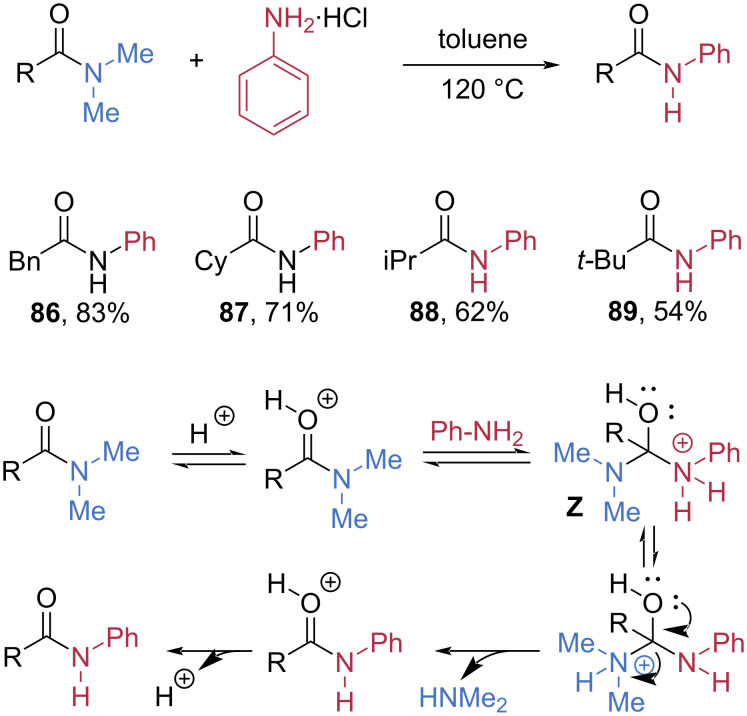
Catalyst- and reagent-free transamidation of amide using aniline hydrochloride salt.

The Karpoormath group discovered that ammonium salts can serve as nucleophiles for the transamidation of amides without the need for any catalyst, base, or additional reagent (Scheme 15) [63]. Aniline hydrochloride efficiently underwent transamidation with *N,N-*dimethylamides possessing primary, secondary, and tertiary alkyl moieties at the α-position, furnishing the corresponding amides **86**–**89** in good to high yields. The efficiency of transamidation decreased with increasing steric congestion. Mechanistically, the carbonyl group is first activated by hydrogen chloride acting as a Lewis acid, enabling nucleophilic attack by the amine to form a tetrahedral intermediate **Z**. Subsequent intramolecular hydrogen shift, followed by elimination of dimethylamine, affords the protonated transamidation product.

**Scheme 16:**
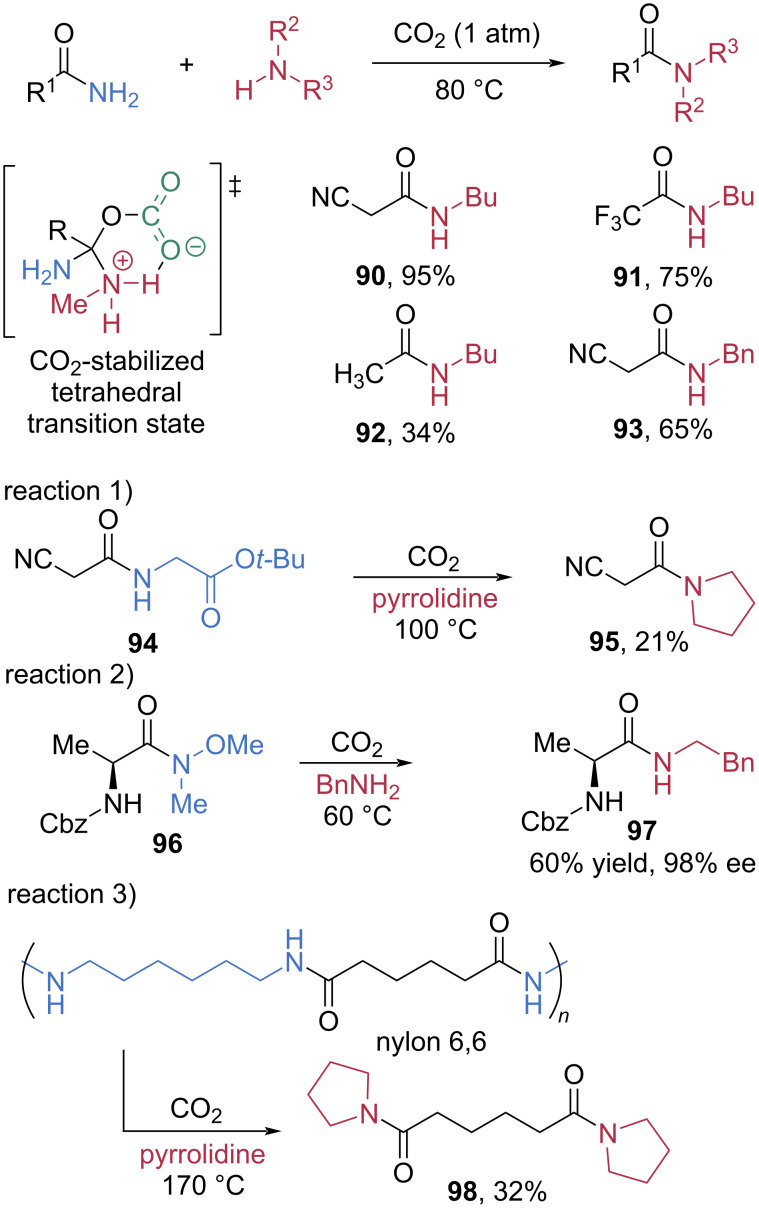
CO_2_-catalyzed transamidation of amides.

In 2021, Lee et al. discovered that carbon dioxide (CO_2_) can catalyze transamidation reactions of amides by activating and stabilizing the tetrahedral transition state, which is a key intermediate (Scheme 16) [64]. Under a CO_2_ atmosphere, electron-deficient primary amides underwent smooth transamidation with butylamine to afford amide products **90** and **91** in good to excellent yields. In the case of electron-enriched primary amides **92**, the yield was decreased. Benzylamine was also a competent nucleophile, providing the corresponding amide **93** in moderate yield. Significantly, this CO_2_-mediated strategy also enabled chemoselective peptide bond cleavage. The glycine residue in **94** was replaced by pyrrolidine in the presence of an ester moiety, albeit in modest yield (**95** = 21%). Notably, Weinreb amides underwent even more efficient transamidation under milder conditions. Chiral alanine-derived Weinreb amide **96** reacted with benzylamine to provide **97** in 60% yield and 98% ee. Furthermore, nylon 6,6 was cleanly depolymerized to its monomer **98** in 32% yield.

**Scheme 17:**
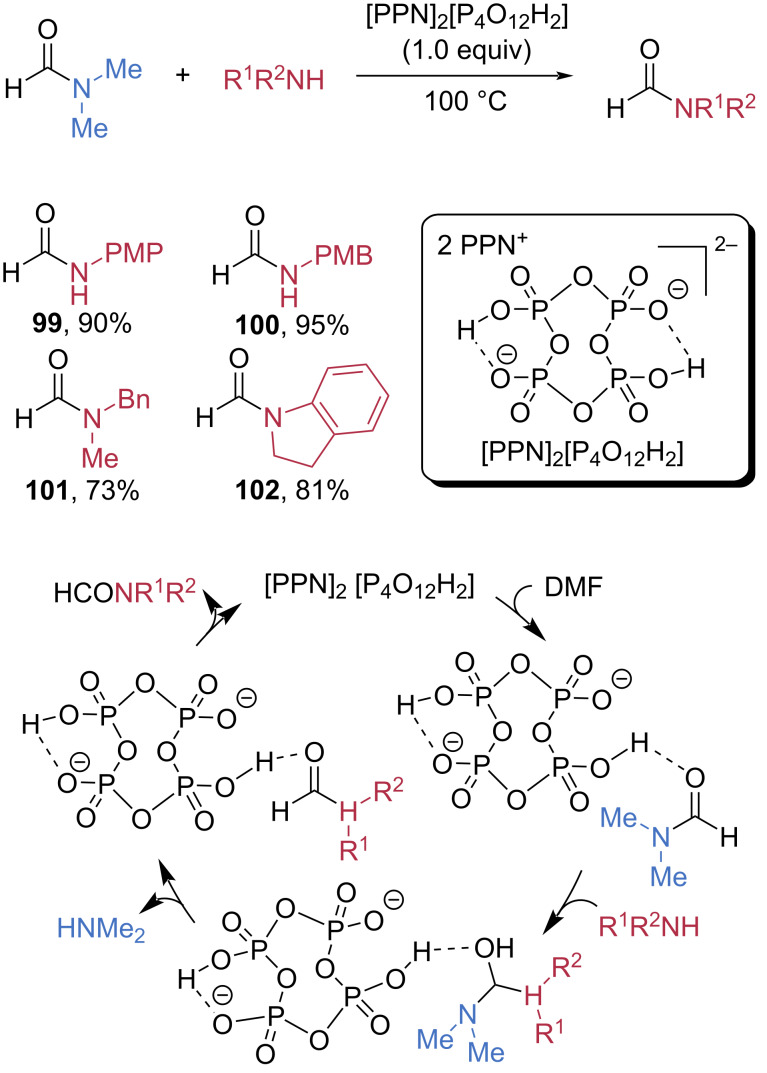
Transamidation of formamides using cyclic dihydrogen tetrametaphosphate.

The Mukherjee group employed dihydrogen tetrametaphosphate as a Lewis acid to activate formamides for transamidation (Scheme 17) [65]. In the presence of one equivalent of [PPN]_2_[P_4_O_12_H_2_], DMF reacted smoothly with *p*-methoxyphenyl (PMP) amine, *p*-methoxybenzyl (PMB) amine, *N*-benzyl-*N-*methylamine, and indole, generating the corresponding amides **99**–**102** in good to excellent yields. Based on kinetic studies, the authors proposed a plausible mechanism in which the dihydrogen tetrametaphosphate dianion activates the amide carbonyl, thereby facilitating nucleophilic attack and subsequent transamidation.

**Scheme 18:**
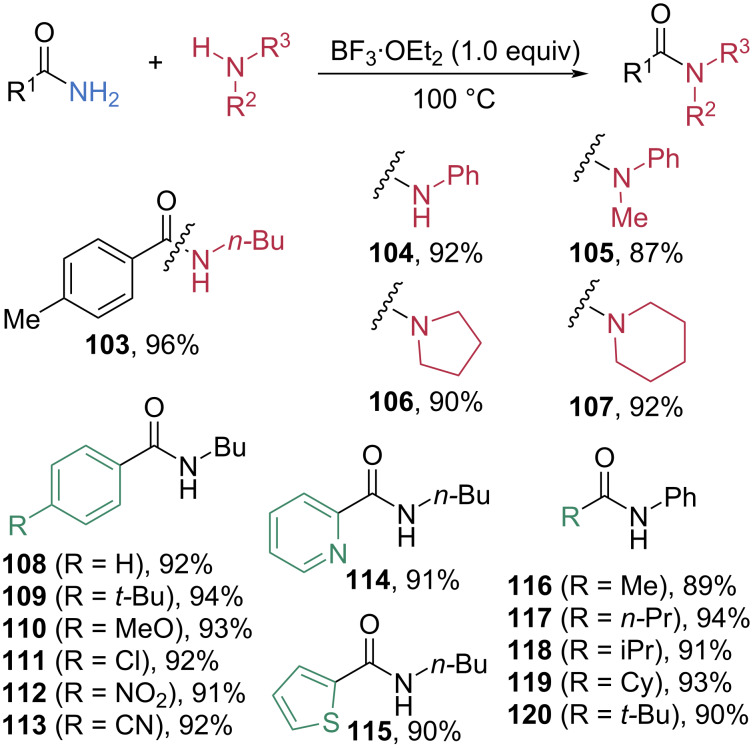
BF_3_·OEt_2_-mediated transamidation of primary amides.

Boron complexes, among the most commonly used non-metallic Lewis acids, are effective activators for amide C–N bond cleavage, as demonstrated in the esterification developed by Dou et al. (Scheme 12) [60]. In 2024, Li et al. employed boron trifluoride etherate (BF_3_·OEt_2_) to promote transamidation of amides (Scheme 18) [66]. The reaction of primary *p*-methylbenzamide with butylamine in the presence of one equivalent of BF_3_·OEt_2_ afforded *N-*butyl-*p*-methylbenzamide (**103**) in 96% yield. Less nucleophilic anilines and bulky secondary amines also reacted smoothly, affording the corresponding amide products **104**–**107** in high to excellent yields. With respect to the R¹ substituent, phenyl, electron-rich phenyl, electron-poor phenyl, and heteroaryl groups were all well tolerated, affording the corresponding amides **108**–**115** in excellent yields. Beyond benzamides, a variety of α-alkylamides possessing primary, secondary, and tertiary moieties also underwent efficient transamidation with aniline, furnishing the amide products **116**–**120** in excellent yields. This transformation proceeds through a mechanism analogous to that proposed in Scheme 12.

#### Halogenation

**Scheme 19:**
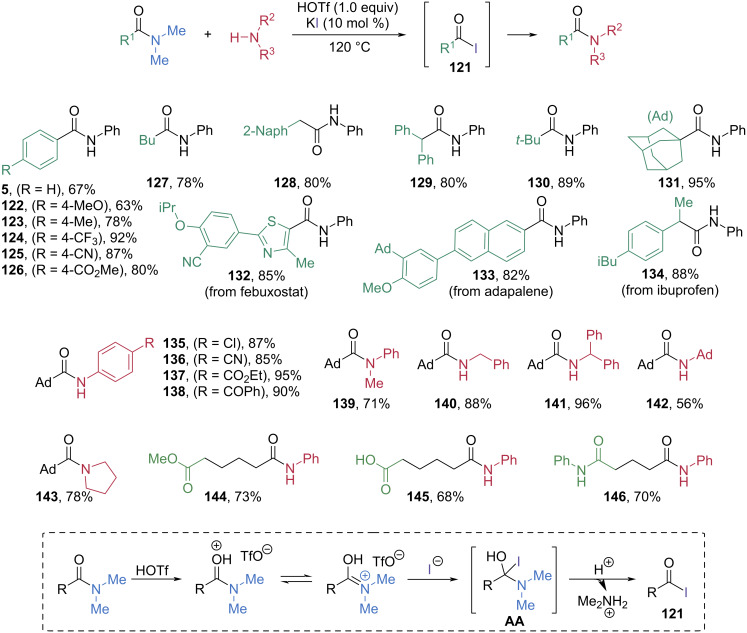
Acyl iodide intermediate **121** generation from amides for the transamidation using HOTf and KI.

In 2022, Szostak and Chen developed an elegant and highly chemoselective transamidation of non-activated tertiary amides via in-situ generation of an acyl iodide intermediate **121** (Scheme 19) [67]. In the presence of a catalytic amount of KI and one equivalent of HOTf, *N,N*-dimethylbenzamide **3** was smoothly converted into a benzoyl iodide intermediate, which underwent nucleophilic acyl substitution with aniline to give benzanilide **5** in 67% yield. Substrate screening demonstrated the broad applicability of this protocol. Various arylamides possessing electron-donating and electron-withdrawing substituents, and alkylamides having primary, secondary, and tertiary alkyl moieties at the α-position, were also suitable substrates, producing the corresponding amides **122**–**131** in high yields via successful generation of the acyl iodide intermediates **121**. Bioactive molecule-derived amides with complex structures smoothly underwent acyl iodide generation, thereby furnishing the products **132**, **133**, and **134** in 85%, 82%, and 88% yield, respectively, via in-situ amidation with aniline. The scope of amine nucleophiles was broad as well. Anilines, primary amines, and secondary amines all furnished the corresponding amides **135**–**143** in high yields, though the sterically hindered 1-adamantylamine afforded the corresponding amide **142** in a moderate 56% yield. Notably, the tertiary amide moiety was cleaved with excellent chemoselectivity even in the presence of other sensitive functional groups, including esters (**144**), carboxylic acids (**145**), and primary anilides (**146**). Mechanistically, the tertiary amide is first protonated and attacked by iodide to generate a tetrahedral intermediate **AA**. Subsequently, removal of *N,N*-dimethylamine as its salt generates the acyl iodide intermediate **121**. The amide product is then formed by acyl substitution of the acyl iodide with the amine nucleophile.

**Scheme 20:**
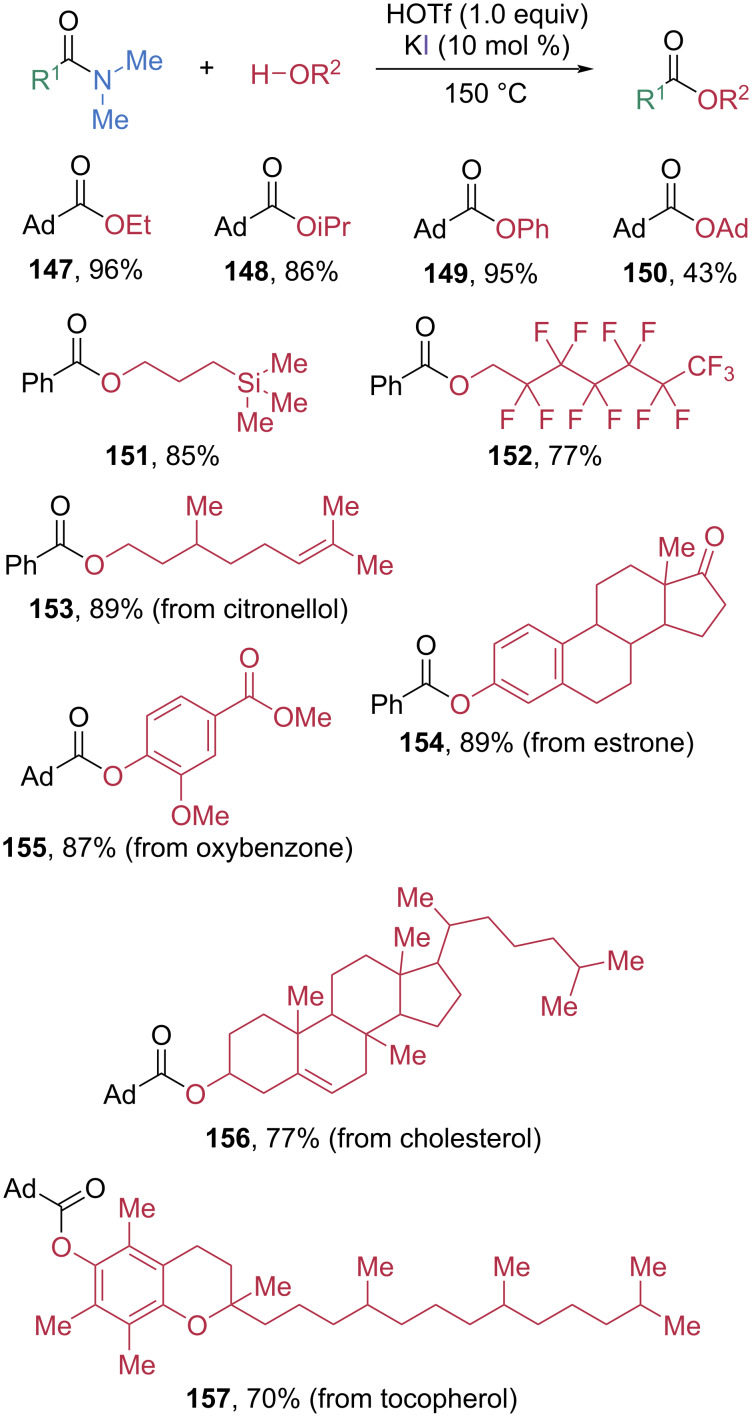
Esterification of *N,N*-dimethyl amides via electrophilic generation of acyl iodide intermediates.

Since the amidation proceeds through acyl iodide intermediates, a wide range of carboxylic acid derivatives can be generated simply by varying the nucleophile. For example, replacing the amine nucleophiles with alcohols enables esterification of the amide (Scheme 20) [68]. Using ethanol, isopropanol, and phenol as nucleophiles, the sterically demanding α-adamantyl *N,N*-dimethylamide was efficiently converted into the corresponding esters **147**–**152** in high to excellent yields, whereas the extremely bulky tertiary alcohol 1-adamantanol afforded the ester product **150** in moderate 43% yield. Notably, this esterification protocol is compatible with structurally complex nucleophiles, enabling late-stage modification of drug-derived amides and bioactive OH-containing molecules **153**–**157**.

### Base-promoted activation assisted by alkali metal counter cations

Although strong bases can promote nucleophilic transformations of amides, such reactions have traditionally required harsh conditions [69–70]. Recently, however, the role of alkali metal counter cations in facilitating acyl substitution has been recognized, enabling efficient direct transamidation under mild conditions.

**Scheme 21:**
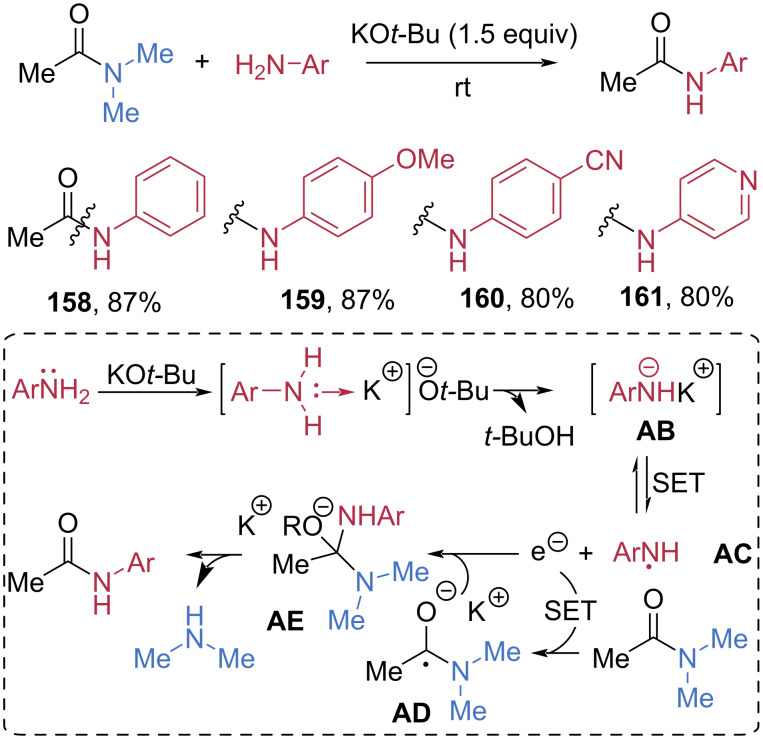
Transamidation of DMAc promoted by KO*t*-Bu.

In 2019, Dash et al. reported a transition-metal-free transamidation of DMAc under mild conditions (Scheme 21) [71]. In the presence of 1.5 equivalents of potassium *tert*-butoxide (KO*t*-Bu) as a base, DMAc underwent smooth transamidation with aniline furnishing acetylanilide **158** in 87% yield. A wide range of arylamines bearing electron-donating or electron-withdrawing substituents, as well as heteroarylamines, were also competent nucleophiles, delivering the corresponding amides **159**–**161** in good yields. EPR studies suggested a single-electron-transfer (SET)-driven mechanism. Coordination of the arylamine to the potassium cation followed by deprotonation generates potassium amide salt **AB**. A SET process forms amine radical **AC**, which then transfers an electron to the amide, generating radical anion **AD**. Coupling of **AC** and **AD** forms a tetrahedral intermediate **AE**, and loss of dimethylamine affords the transamidated products. One year later, Gu et al. developed a related transamidation protocol mediated by sodium *tert*-butoxide (NaO*t*-Bu) under neat conditions [72].

**Scheme 22:**
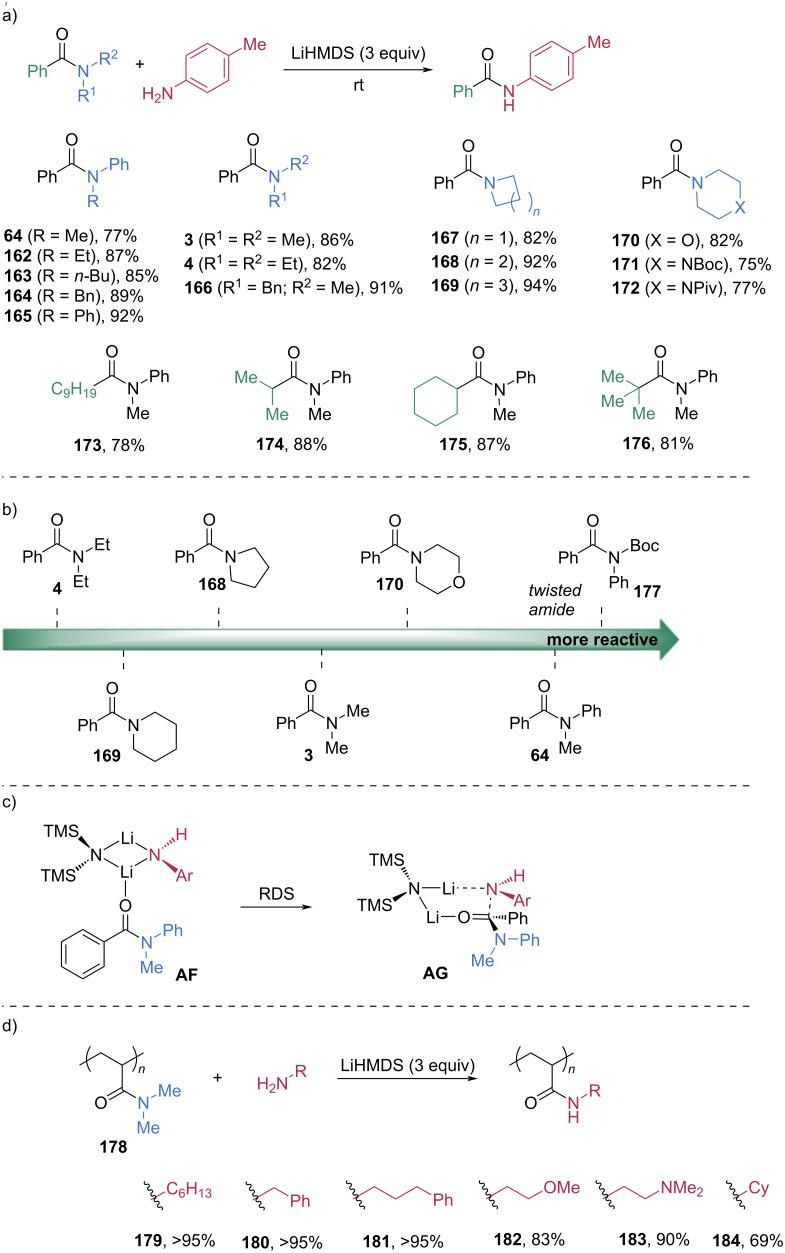
a) LiHMDS-mediated transamidation of tertiary amides. b) Computed reactivities of selected amides. c) Rate-determining step of the LiHMDS-mediated transamidation. d) LiHMDS-mediated transamidation of an acrylamide polymer.

In 2019, Szostak et al. reported an elegant and chemoselective transamidation of unactivated tertiary amides under transition-metal-free conditions [73]. Lithium hexamethyldisilazide (LiHMDS) was used to activate the amide, enabling smooth cleavage of the C–N bond even in sterically hindered substrates (Scheme 22a). Using *p*-methylaniline as a nucleophile in the presence of three equivalents of LiHMDS, *N*-alkyl- and *N*-arylanilides were efficiently cleaved to afford the corresponding amides **64** and **162**–**165** in high yields. A broad range of *N,N*-dialkylamides, cyclic amides, and heterocyclic amides were also compatible, all delivering amide products **3**, **4**, and **166**–**172** in good to excellent yields. Additionally, α-alkyl-substituted amides were effectively transformed into the corresponding amide products **173**–**176** in high yields under the optimal conditions. DFT studies provided insight into the relative reactivities of diverse amide substrates (Scheme 22b). *N*-Methylbenzanilide (**64**) exhibited lower reactivity than the twisted *N*-Boc-*N*-phenylbenzamide (**177**) but was more reactive than *N,N*-dialkylamides **3**, **4**, **168**, **169**, and **170**. Computational analysis also revealed that the intramolecular nucleophilic attack of intermediate **AF** through transition state **AG** constitutes the rate-determining step (Scheme 22c). Leveraging this LiHMDS-mediated transamidation strategy, Sumerlin et al. subsequently demonstrated efficient post-polymerization modification of acrylamide-based polymers **178** via direct transamidation (Scheme 22d) [74].

### Installation of activating unit

For the efficient transformation of amide bonds, an activating unit can be introduced on the amide nitrogen to assist the cleavage of the amide C–N bond. This approach facilitates not only an improvement of the inherent low reactivity but also the chemoselective transformation of target amides (those possessing the activating unit) under specific reaction conditions.

**Scheme 23:**
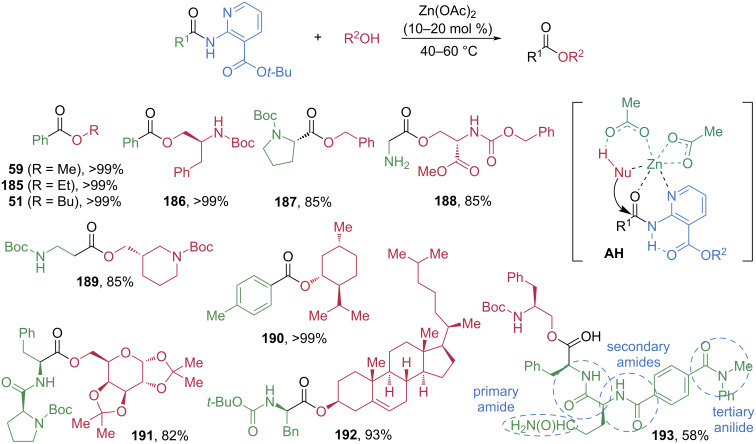
Zn-catalyzed chemoselective cleavage of amides directed by *tert*-butyl nicotinate.

The Maes group introduced a *tert*-butyl nicotinate (*N-t*-Bu *nic*) moiety as a directing group to coordinate with transition metals, thereby enhancing electrophilicity and promoting esterification (Scheme 23) [75]. In the presence of a Zn catalyst, the amide is activated through bidentate chelation, as depicted in the proposed transition state **AH**, which facilitates nucleophilic attack of alcohols at the carbonyl center and subsequent C–N bond cleavage. Under the optimized conditions, *tert*-butyl benzamidonicotinate reacted smoothly with methanol, ethanol, and butanol to afford alkyl benzoates **59**, **185**, and **51** in essentially quantitative yields. A key advantage of this protocol is its applicability to amino acid- and peptide-derived amides. Complex alcohols were successfully employed, producing the corresponding esters **186**–**192** in high yields without any detectable racemization or epimerization. Notably, the chemoselective esterification of a complex peptide, containing four distinct amide bonds and the *tert*-butyl nicotinate directing group, delivered the ester product **193** in 58% yield.

**Scheme 24:**
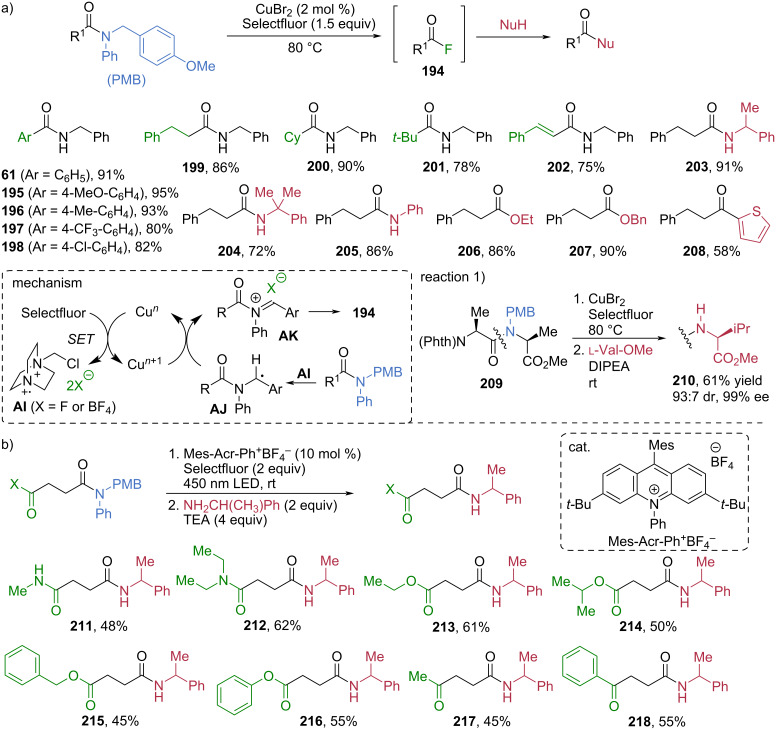
Chemoselective cleavage of *N*-PMB anilide for transamidation via acyl fluoride **194** generation. a) Cu(II) bromide/Selectfluor hybrid system for cleavage of *N*-PMB anilide. b) Metal-free photoredox catalysis for cleavage of *N*-PMB anilide.

The *p*-methoxybenzyl (PMB) group was recognized as an efficient activating unit for the selective cleavage of tertiary anilides in 2020 [76]. In the presence of CuBr_2_ and Selectfluor as an electrophilic fluorine source, the C–N bond of *N*-PMB-substituted anilides undergoes smooth oxidative cleavage to form the corresponding acyl fluoride intermediate **194**, thereby facilitating transamidation via in-situ addition of an amine nucleophile (Scheme 24a). A broad range of aryl-substituted *N*-PMB anilides with electron-donating, electron-withdrawing, or neutral substituents were effectively converted to the corresponding amides **61** and **195**–**198** in high to excellent yields. Alkyl-substituted *N*-PMB anilides, including primary, secondary, and tertiary alkyl moieties, were also suitable substrates, each furnishing the amides **199**–**201** in good to high yields. Even the less reactive conjugated *N*-PMB anilides underwent transamidation to give the amide product **202** in 75% yield. Once an acyl fluoride intermediate is generated, a variety of nucleophiles could be efficiently employed in the subsequent transformation. Bulky primary amines and aniline, whose nucleophilicities are diminished by steric congestion and low electron density, respectively, still reacted smoothly with the in-situ-generated acyl fluorides, furnishing the corresponding hindered amides and anilides **203**–**205** in high yields. Likewise, esters **206** and **207** and ketones **208** were efficiently obtained by treating the acyl fluoride intermediates **194** with alcohols as *O*-nucleophiles and thiophene as a *C*-nucleophile, respectively. Mechanistic studies support a radical pathway. Selectfluor undergoes a single-electron transfer (SET) with Cu(II) to generate the dicationic radical species **AI**, which abstracts a hydrogen atom from the PMB group to form a benzylic radical intermediate **AJ**. A subsequent SET process generates an acyliminium intermediate **AK**, which then decomposes to furnish the acyl fluorides **194**. This strategy provided chemoselective peptide modification. For example, the amide bond possessing the PMB unit in dipeptide **209** (*N*-Phth-ʟ-Ala-*N*-PMB-ʟ-Ala-OMe) was selectively activated and cleaved, generating the corresponding acyl fluoride intermediate. The in-situ amidation of the resulting acyl fluoride with ʟ-valine methyl ester produced **210** in 61% yield with 93:7 dr and 99% ee.

In 2024, a metal-free photoredox catalytic method for the transamidation of PMB anilides was developed [77]. This protocol emphasizes chemoselective cleavage and transformation of the target amide in the presence of more reactive functional groups (Scheme 24b). Using 9-mesityl-3,6-di-*tert*-butyl-10-phenylacridinium tetrafluoroborate (Mes-Acr-Ph^+^BF_4_^−^) as the photoredox catalyst under 450 nm LED irradiation, the PMB anilide unit was chemoselectively activated and cleaved in the presence of secondary or tertiary amides to generate the corresponding acyl fluoride intermediate. Subsequent in-situ amidation with 2-methylbenzylamine furnished diamide products **211** and **212** in 48% and 62% yields, respectively. Amido esters and amido ketones were likewise converted to the corresponding products **213**–**218** in moderate yields. Remarkably, in all cases, the otherwise bulky and less reactive amide was preferentially cleaved over less-hindered and more electrophilic functionalities, demonstrating unusual and highly valuable chemoselectivity in amide activation and transformation.

## Conclusion

This review has highlighted major advances from the past decade in the activation, cleavage, and transformation of non-activated amides. Although the amide bond is fundamental to chemistry and biology, its exceptional resonance stabilization renders it remarkably resistant to nucleophilic acyl substitution, making its selective transformation a long-standing challenge in organic synthesis. While strategies for activating twisted amides, bearing electron-withdrawing and sterically demanding substituents on nitrogen, are now well established, general and efficient methods for modifying conventional, non-activated amides have only recently begun to mature. To address the inherent stability of these classical amides, several complementary activation strategies have emerged. Transition-metal Lewis acids, long recognized for their ability to enhance carbonyl electrophilicity through coordination, continue to provide powerful platforms for amide functionalization. Electrophilic activation, particularly via in-situ generation of keteniminium or related highly reactive intermediates, has enabled transamidation, esterification, and halogenation under metal-free conditions. Strong bases, especially when coupled with cooperative alkali metal counter cations, have expanded the scope of amide C–N bond cleavage under mild conditions. In parallel, the installation of activating groups on the amide nitrogen has enabled chemoselective cleavage, opening the door to late-stage transformations of target amides even in molecules bearing other reactive functional groups.

Collectively, these advances provide a rapidly expanding toolbox for achieving amide bond activation under increasingly mild, selective, and practical conditions. We anticipate that the concepts summarized here will not only deepen mechanistic understanding of amide reactivity but also inspire the future development of broadly applicable, efficient, and environmentally sustainable strategies for amide transformation.

## Data Availability

Data sharing is not applicable as no new data was generated or analyzed in this study.
